# Antihypertensive Medication Classes Used among Medicare Beneficiaries Initiating Treatment in 2007–2010

**DOI:** 10.1371/journal.pone.0105888

**Published:** 2014-08-25

**Authors:** Shia T. Kent, Daichi Shimbo, Lei Huang, Keith M. Diaz, Meredith L. Kilgore, Suzanne Oparil, Paul Muntner

**Affiliations:** 1 Department of Epidemiology, University of Alabama at Birmingham, Birmingham, Alabama, United States of America; 2 Center for Behavioral Cardiovascular Health, Department of Medicine, Columbia University Medical Center, New York, New York, United States of America; 3 Department of Health Care Organization and Policy, University of Alabama at Birmingham, Birmingham, Alabama, United States of America; 4 Department of Medicine, Division of Cardiovascular Disease, University of Alabama at Birmingham, Birmingham, Alabama, United States of America; Cleveland Clinic Lerner Research Institute, United States of America

## Abstract

**Background:**

After the 2003 publication of the Seventh Report of the Joint National Committee on Prevention, Detection, Evaluation, and Treatment of High Blood Pressure (JNC 7) guidelines, there was a 5–10% increase in patients initiating antihypertensive medication with a thiazide-type diuretic, but most patients still did not initiate treatment with this class. There are few contemporary published data on antihypertensive medication classes filled by patients initiating treatment.

**Methods and Findings:**

We used the 5% random Medicare sample to study the initiation of antihypertensive medication between 2007 and 2010. Initiation was defined by the first antihypertensive medication fill preceded by 365 days with no antihypertensive medication fills. We restricted our analysis to beneficiaries ≥65 years who had two or more outpatient visits with a hypertension diagnosis and full Medicare fee-for-service coverage for the 365 days prior to initiation of antihypertensive medication. Between 2007 and 2010, 32,142 beneficiaries in the 5% Medicare sample initiated antihypertensive medication. Initiation with a thiazide-type diuretic decreased from 19.2% in 2007 to 17.9% in 2010. No other changes in medication classes initiated occurred over this period. Among those initiating antihypertensive medication in 2010, 31.3% filled angiotensin-converting enzyme inhibitors (ACE-Is), 26.9% filled beta blockers, 17.2% filled calcium channel blockers, and 14.4% filled angiotensin receptor blockers (ARBs). Initiation with >1 antihypertensive medication class decreased from 25.6% in 2007 to 24.1% in 2010. Patients initiated >1 antihypertensive medication class most commonly with a thiazide-type diuretic and either an ACE-I or ARB.

**Conclusion:**

These results suggest that JNC 7 had a limited long-term impact on the choice of antihypertensive medication class and provide baseline data prior to the publication of the 2014 Evidence-Based Guideline for the Management of High Blood Pressure in Adults from the Panel Members Appointed to the Eighth Joint National Committee (JNC 8).

## Introduction

In 2002, the Antihypertensive and Lipid-Lowering Treatment to Prevent Heart Attack Trial (ALLHAT) found that in a hypertensive population with at least one additional coronary heart disease (CHD) risk factor, randomization to chlorthalidone (thiazide-type diuretic), amlodpine (calcium channel blocker [CCB]), or lisinopril (angiotensin-converting enzyme inhibitor [ACE-I]) was associated with similar rates of coronary heart disease outcomes [1].Chlorthalidone was associated with a lower risk of heart failure, a secondary outcome. Shortly after the publication of the main results of ALLHAT, the Seventh Report of the Joint National Committee on the Prevention, Detection, Evaluation, and Treatment of High Blood Pressure (JNC 7) published guidelines for the prevention and treatment of hypertension [2].

Based in part on the comparative effectiveness results shown in ALLHAT, and due to their lower cost, JNC 7 supported the use of thiazide-type diuretics as first line therapy for those without a compelling indication for treatment with another antihypertensive drug class. For patients with compelling indications (e.g. chronic kidney disease [CKD]), use of other classes of antihypertensive medication was recommended. Several studies have examined the impact of ALLHAT and JNC 7 on classes of antihypertensive medication being filled by patients initiating treatment [3]–[5]. These studies reported a 5–10% increase in the initiation of antihypertensive treatment with thiazide-type diuretics after the publication of ALLHAT and JNC 7. However, the majority of patients initiated antihypertensive medication with other drug classes during this period, indicating that JNC 7 guidelines had a limited impact on the choice of drug class. There are few recent data on whether adherence to JNC 7 drug class recommendations has increased or decreased over time. While it is possible that thiazide-type diuretic usage has increased, ACE-Is and CCBs have since decreased in cost [6], [7]. Additionally, subsequent trials comparing antihypertensive drug classes on cardiovascular outcomes and a meta-analysis has suggested no substantial differences exist between ACE-Is, angiotensin receptor blockers (ARBs), CCBs, and thiazide-type diuretics [8]–[12].Also, guidelines and policy statements from various associations and institutions published since JNC 7 have not universally recommended thiazide-type diuretics for patients initiating treatment [13]–[15]. These factors could influence current trends in choices of first-line therapy among US adults.

The long-term impact of JNC 7 is of relevance to the current and future dissemination of guidelines, such as the recently published 2014 Evidence-Based Guideline for the Management of High Blood Pressure in Adults from the Panel Members Appointed to the Eighth Joint National Committee (JNC 8) [16]. Contemporary data can provide a baseline documentation of whether patients are initiating antihypertensive medication with the classes recommended by JNC 8. Therefore, the goal of the present study was to examine the classes of antihypertensive medication being filled among US Medicare beneficiaries initiating treatment. Additionally, we evaluated the association between patient factors, including demographics and comorbidities, with the initiation of antihypertensive medication classes. To accomplish these goals, we analyzed the 5% random sample of US Medicare beneficiaries initiating antihypertensive medication between 2007 through 2010.

## Methods

We conducted a retrospective cohort study of Medicare beneficiaries using the 2006–2010 national 5% random sample from the Centers for Medicare and Medicaid Services (CMS). Medicare is a US federal benefit program that provides health insurance to individuals 65 years of age and older, on disability, or who have end-stage renal disease, through either individual fee-for-service claims or contracts with health care organizations. Specific data used for the current analyses include claims from Medicare fee-for-service Parts A (in-patient), B (out-patient) and D (prescription drug). These data provide Medicare claims data linked by beneficiary across the continuum of care. The CMS and the Institutional Review Board at the University of Alabama at Birmingham approved the study. Beneficiary records were anonymized and de-identified prior to analysis.

The current analysis included Medicare beneficiaries who initiated antihypertensive medication in 2007, 2008, 2009 or 2010. The initial eligibility criterion was defined by filling an antihypertensive medication between January 1, 2007 and December 25, 2010. The date of each beneficiary's first fill for an antihypertensive medication in a calendar year was used as their index fill date. December 25, 2010 rather than December 31, 2010 was chosen for the end of the study period to accommodate a 6 day period to identify additional antihypertensive medication classes filled among patients initiating treatment (see below). To facilitate the identification of prevalent antihypertensive medication users for exclusion from our analyses, beneficiaries were required to have continuous full Medicare coverage (traditional Medicare Parts A and B fee-for-service and Part D coverage) and reside in the 50 United States or Washington DC for the 365 day period preceding the index fill date. This period is referred to as the “look-back” period. To capture medications filled as part of the initial antihypertensive regimen, we required beneficiaries to have full Medicare coverage for 6 days following their index fill date.

To increase the likelihood that the antihypertensive medications were filled to lower blood pressure, we limited the sample to beneficiaries with hypertension, defined by ≥2 outpatient physician evaluation and management claims, ≥7 days apart, with International Classification of Diseases, 9th Revision (ICD-9) diagnoses of 401.x (malignant, benign or unspecified essential hypertension) during the look-back period. We excluded beneficiaries who were prevalent users of antihypertensive medication defined by any antihypertensive medication fills during the 365 day look-back period. Also, we excluded beneficiaries who were <65 years of age at the start of the 365 day look-back period or ≥110 years of age on the date of the index antihypertensive medication fill. To examine possible time trends, we initially created separate yearly cohorts of beneficiaries initiating antihypertensive medication in 2007, 2008, 2009, and 2010. A beneficiary could potentially be counted in multiple years if they had an antihypertensive medication fill more than 365 days prior to the index fill. In subsequent analyses, we pooled the data to create a single cohort that only included each beneficiary's first antihypertensive medication fill in the 2007–2010 time period. A CONSORT diagram showing the inclusion/exclusion of Medicare beneficiaries is provided in Figure S1.

### Antihypertensive medication fills

Claims for antihypertensive medications were identified in the Medicare Part D file. Antihypertensive medications filled from the index fill date through the next 6 days were considered to be initiated as part of the same regimen. Antihypertensive medications were grouped into drug classes using classifications from JNC 7, with newer medications identified by review of the study authors (D.S., S.O.). Pills containing two antihypertensive medication classes were considered combination medications and patients were considered to initiate both classes.

### Covariates

*A priori*-selected covariates were used to study the characteristics of Medicare beneficiaries initiating each class of antihypertensive medication. These included age, sex, race/ethnicity, Medicaid buy-in (a measure of poverty) for the entire look-back period, and comorbid conditions that may be considered compelling indications for being prescribed certain antihypertensive medication classes (diabetes, coronary heart disease [CHD], stroke, CKD, and heart failure) [2]. Age, sex, race/ethnicity, and Medicaid buy-in were defined using the Medicare beneficiary enrollment file. Comorbid conditions were defined using claims during the look-back period and previously published algorithms (Appendix S1).

### Statistical analyses

Characteristics of Medicare beneficiaries initiating antihypertensive medications were calculated by calendar year of initiation. For each calendar year, the percentages initiating each antihypertensive medication class, initiating >1 class, and initiating antihypertensive treatment with a combination pill were calculated. Among those who initiated therapy with >1 antihypertensive medication class, we examined the pairs of classes that were initiated. P-values for trends across calendar year were calculated in Poisson regression models with calendar year as a continuous variable. Using the pooled 2007–2010 cohort, we calculated the distributions of antihypertensive medication classes initiated for the overall population and in subgroups defined by gender, race/ethnicity (black, white, Hispanic, Asian, or other), the presence or absence of each compelling indication (diabetes, CHD, stroke, CKD, and heart failure), and with any or no compelling indication, separately. We then calculated the risk ratios for initiating each antihypertensive medication class using Poisson regression and sandwich estimators. Initiating each antihypertensive medication class was evaluated in a separate regression model with the comparison group consisting of individuals initiating an antihypertensive medication regimen without that class (e.g., initiating with an ACE-I versus initiating treatment with any other antihypertensive medication classes). Adjusted risk ratios for initiating each antihypertensive medication class were calculated for calendar year of initiation, age, gender, race/ethnicity, Medicaid buy-in, diabetes, CHD, CKD, and heart failure. Adjusted risk ratios for initiating each antihypertensive medication class were also calculated among Medicare beneficiaries without any compelling indications. Lastly, we calculated the percent of beneficiaries in the pooled 2007–2010 cohort who initiated antihypertensive medication with classes recommended by the 2014 JNC 8 guideline. Among beneficiaries without CKD, recommended classes include a thiazide-type diuretic or a CCB for blacks and a thiazide-type diuretic, CCB, ACE-I, or ARB for non-blacks [16]. For beneficiaries with CKD, recommended classes include an ACE-I or an ARB. Analyses were conducted using SAS 9.3 (SAS Institute, Cary, NC).

## Results

In 2007, 2008, 2009, and 2010 there were 7,456, 8,769, 8,575, and 8,719 eligible Medicare beneficiaries, respectively, who initiated antihypertensive medication. Characteristics of Medicare beneficiaries in the 5% sample who initiated antihypertensive medications in 2007, 2008, 2009 and 2010 are provided in Table 1. The mean age was 77 years, approximately 80% were white and 37% to 41% were men. Diabetes was the most prevalent comorbid condition, followed by a history of CHD. In each year, approximately 50% of Medicare beneficiaries who initiated antihypertensive medication had a history of diabetes, CHD, stroke, CKD or heart failure. Beneficiaries initiating therapy in later study years were younger and more likely to be male, white, and have diabetes and CKD, and less likely to be black and have had a stroke or heart failure. The presence of any compelling indication (diabetes, CHD, stroke, CKD, and heart failure) for being prescribed certain antihypertensive medication classes was more common in recent study years.

**Table 1:** Characteristics of Medicare beneficiaries initiating antihypertensive medication, by calendar year.

	Calendar year				
Characteristic	2007 (n = 7456)	2008 (n = 8769)	2009 (n = 8575)	2010 (n = 8719)	p-value for trend
Age, years	77.0 (7.6)	77.1 (7.7)	76.8 (7.6)	76.7 (7.6)	<0.001
Male	2753 (36.9%)	3310 (37.7%)	3368 (39.3%)	3540 (40.6%)	<0.001
Race/ethnicity					
White	5933 (79.6%)	7117 (81.2%)	7015 (81.8%)	7189 (82.5%)	<0.001
Black	826 (11.1%)	914 (10.4%)	771 (9.0%)	775 (8.9%)	<0.001
Hispanic	270 (3.6%)	301 (3.4%)	307 (3.6%)	283 (3.2%)	0.27
Asian	220 (3.0%)	220 (2.5%)	274 (3.2%)	252 (2.9%)	0.48
Other	207 (2.8%)	217 (2.5%)	208 (2.4%)	220 (2.5%)	0.32
Medicaid buy-in	2007 (26.9%)	1977 (22.5%)	1916 (22.3%)	1857 (21.3%)	<0.001
Diabetes	1966 (26.4%)	2329 (26.6%)	2346 (27.4%)	2463 (28.2%)	0.003
Coronary heart disease	1310 (17.6%)	1496 (17.1%)	1524 (17.8%)	1516 (17.4%)	0.90
Stroke	583 (7.8%)	661 (7.5%)	583 (6.8%)	600 (6.9%)	0.006
Chronic kidney disease	666 (8.9%)	840 (9.6%)	905 (10.6%)	1041 (11.9%)	<0.001
Heart failure	861 (11.5%)	941 (10.7%)	867 (10.1%)	925 (10.6%)	0.03
Any compelling indication†	3585 (48.1%)	4260 (48.6%)	4207 (49.1%)	4394 (50.4%)	0.002

Numbers in table are mean (standard deviation) for age and number (percent) for other characteristics.

† Others include alpha blockers, central acting agents, direct vasodilators, aldosterone receptor blockers, and renin inhibitors.

ACE-Is were the most commonly initiated antihypertensive medication class in each year (Table 2). The next most commonly initiated medication classes were beta blockers, thiazide-type diuretics, CCBs, and ARBs. Between 24% and 26% of the cohort initiated antihypertensive medication with >1 class and 11% to 12% initiated treatment with a combination pill. Among those who initiated multiple classes (either with a combination pill or as separate pills), a thiazide-type diuretic with an ACE-I or a thiazide-type diuretic with an ARB were mostly commonly initiated (Table S1). Other antihypertensive medication pairs commonly initiated were an ACE-I with a beta blocker or an ACE-I with a CCB.

**Table 2:** Antihypertensive medication classes initiated among Medicare beneficiaries, by calendar year.

	Calendar year				
Antihypertensive medication class	2007 (n = 7456)	2008 (n = 8769)	2009 (n = 8575)	2010 (n = 8719)	p-value for trend
Renin angiotensin system blockers	3416 (45.8%)	3919 (44.7%)	3914 (45.6%)	3977 (45.6%)	0.82
ACE-inhibitors	2380 (31.9%)	2662 (30.4%)	2712 (31.6%)	2728 (31.3%)	0.87
Angiotensin receptor blockers	1057 (14.2%)	1280 (14.6%)	1214 (14.2%)	1257 (14.4%)	0.90
All Diuretics	2540 (34.1%)	2999 (34.2%)	2845 (33.2%)	2833 (32.5%)	0.03
Thiazide	1433 (19.2%)	1686 (19.2%)	1616 (18.8%)	1565 (17.9%)	0.03
Loop	929 (12.5%)	1094 (12.5%)	1070 (12.5%)	1090 (12.5%)	0.94
Potassium-sparing	230 (3.1%)	291 (3.3%)	241 (2.8%)	241 (2.8%)	0.06
Beta blockers	2009 (26.9%)	2347 (26.8%)	2290 (26.7%)	2344 (26.9%)	0.93
Calcium channel blockers	1322 (17.7%)	1552 (17.7%)	1442 (16.8%)	1503 (17.2%)	0.21
Others†	417 (5.6%)	481 (5.5%)	489 (5.7%)	527 (6.0%)	0.16
Initiating >1 antihypertensive medication class	1908 (25.6%)	2163 (24.7%)	2090 (24.4%)	2102 (24.1%)	0.03
Initiating a combination pill	899 (12.1%)	993 (11.3%)	963 (11.2%)	966 (11.1%)	0.06

Numbers in table are number (percent).

Numbers of antihypertensive medications initiated are not mutually exclusive; column percentages add to >100%.

Abbreviations: ACE =  angiotensin-converting enzyme.

† Others include alpha blockers, central acting agents, direct vasodilators, aldosterone receptor blockers, and renin inhibitors.

**Figure 1:**
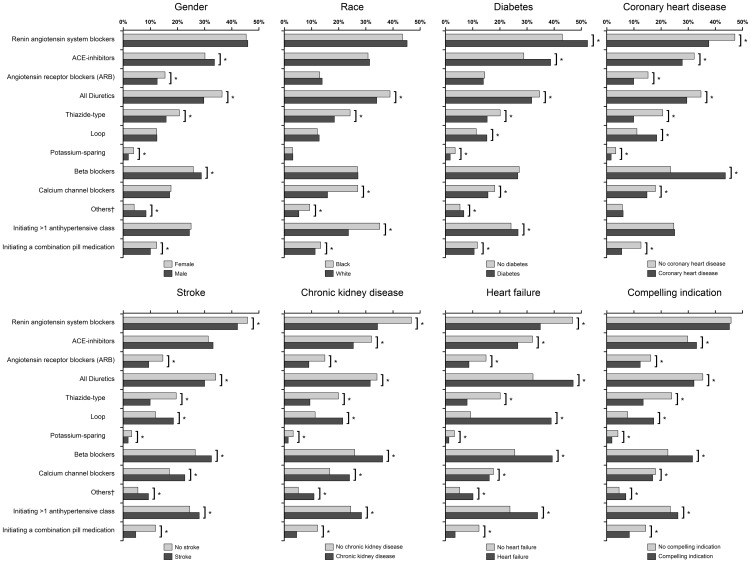
Antihypertensive medication classes initiated among Medicare beneficiaries, by selected covariates. Note: Compelling indication is defined by a beneficiary having diabetes, coronary heart disease, stroke, chronic kidney disease, or heart failure. *p<0.05. ^†^Any compelling indication is defined as having diabetes, coronary heart disease, stroke, chronic kidney disease, or heart failure.

Overall, 32,142 Medicare beneficiaries were included in the pooled 2007–2010 cohort. Figure 1 shows antihypertensive medication classes initiated by beneficiary characteristics in this cohort. Compared to females, males were more likely to initiate antihypertensive medication with an ACE-I or beta blocker and less likely to initiate treatment with an ARB or a thiazide-type diuretic. Compared to whites, blacks were more likely to initiate treatment with a thiazide-type diuretic or CCB. Compared to their counterparts without diabetes, beneficiaries with diabetes were more likely to initiate therapy with an ACE-I and less likely to initiate with a thiazide-type or CCB. Those with CHD, stroke, CKD, or heart failure were less likely than those without each respective condition to initiate treatment with an ARB or with a thiazide-type diuretic and more likely to initiate with a loop diuretic or a beta blocker. Those without any compelling indication (diabetes, CHD, stroke, CKD, or heart failure), compared to those with any compelling indication, were less likely to initiate therapy with an ACE-I or beta blocker and more likely to initiate with an ARB or thiazide-type diuretic. Blacks compared with whites, and those with diabetes, a history of stroke, CKD, and heart failure were more likely to initiate treatment with >1 antihypertensive class.

After multivariable adjustment, there was a decline in the initiation of antihypertensive medication with a thiazide-type diuretic over time (Table 3). Specifically, an 8% reduction (risk ratio 0.92; 95% CI 0.86–0.98) in initiation of antihypertensive medication with a thiazide-type diuretic occurred in 2010, compared with 2007. Drug initiation patterns by each compelling indication were similar to those in unadjusted analyses. Older beneficiaries were less likely to initiate treatment with an ACE-I or thiazide-type diuretic and more likely to initiate with a loop diuretic, beta blocker, or CCB. Males were more likely than females to initiate with an ACE-I and were less likely to initiate with an ARB or a thiazide-type or potassium-sparing diuretic. Compared to females, males were more likely to initiate with an ACE-I and less likely to initiate with and ARB, or a thiazide-type or potassium-sparing diuretic. Compared to whites, blacks were less likely to initiate treatment with an ACE-I or loop diuretic and more likely to initiate treatment with a thiazide-type diuretic or CCB. Compared to whites, Hispanics were more likely to initiate antihypertensive medication with an ACE-I or ARB and less likely to initiate with a loop or potassium-sparing diuretic. Asians, compared with whites were more likely to initiate with an ARB or CCB and less likely to initiate treatment with an ACE-I or loop diuretic. Beneficiaries with a Medicaid buy-in were less likely to initiate treatment with an ARB or a thiazide-type or potassium-sparing diuretic, and were more likely to initiate therapy with a loop diuretic.

**Table 3:** Multivariable adjusted risk ratios and 95% confidence intervals for initiating each antihypertensive medication class among Medicare beneficiaries in 2007–2010.

	Antihypertensive medication class initiated							
Characteristic	ACE-inhibitors (n = 10105)	ARBs (n = 4583)	Thiazide diuretics (n = 6040)	Loop diuretics (n = 3798)	Potassium-sparing diuretics (n = 973)	Beta blockers (n = 8658)	CCBs (n = 593)	>1 class (n = 7961)
Year								
2007	1 (ref)	1 (ref)	ref	1 (ref)	1 (ref)	1 (ref)	1 (ref)	ref
2008	0.95 (0.91–0.99)	1.03 (0.95–1.11)	0.99 (0.93–1.06)	1.03 (0.95–1.11)	1.07 (0.90–1.27)	1.00 (0.95–1.05)	1.00 (0.93–1.07)	0.97 (0.92–1.02)
2009	0.99 (0.95–1.04)	0.99 (0.91–1.06)	0.98 (0.92–1.04)	1.03 (0.95–1.12)	0.93 (0.78–1.11)	1.00 (0.95–1.06)	0.96 (0.90–1.03)	0.97 (0.92–1.03)
2010	0.98 (0.94–1.03)	0.99 (0.92–1.07)	0.92 (0.86–0.98)	1.03 (0.95–1.12)	0.90 (0.75–1.08)	1.01 (0.96–1.06)	0.98 (0.92–1.05)	0.95 (0.90–1.00)
Age								
65–69	1 (ref)	1 (ref)	1 (ref)	1 (ref)	1 (ref)	1 (ref)	1 (ref)	ref
70–74	0.93 (0.89–0.97)	0.95 (0.88–1.03)	0.92 (0.87–0.98)	1.09 (0.98–1.22)	0.99 (0.82–1.18)	1.02 (0.97–1.08)	1.02 (0.94–1.10)	0.90 (0.85–0.95)
75–79	0.85 (0.81–0.90)	0.96 (0.89–1.04)	0.83 (0.77–0.89)	1.41 (1.27–1.56)	0.93 (0.77–1.13)	1.01 (0.96–1.07)	1.16 (1.07–1.25)	0.89 (0.84–0.95)
80–84	0.79 (0.75–0.84)	0.85 (0.78–0.93)	0.78 (0.73–0.84)	1.69 (1.53–1.88)	0.91 (0.74–1.12)	1.09 (1.03–1.15)	1.11 (1.02–1.20)	0.86 (0.81–0.92)
85+	0.73 (0.70–0.78)	0.72 (0.65–0.79)	0.67 (0.62–0.73)	2.23 (2.02 2.46)	0.88 (0.72–1.08)	1.07 (1.01–1.14)	1.20 (1.11–1.30)	0.82 (0.77–0.87)
Male	1.10 (1.06–1.13)	0.84 (0.79–0.88)	0.82 (0.78–0.86)	1.00 (0.95–1.06)	0.55 (0.47–0.64)	1.01 (0.98–1.05)	0.99 (0.94–1.04)	0.95 (0.92–0.99)
Race/ethnicity								
White	1 (ref)	1 (ref)	ref	1 (ref)	1 (ref)	1 (ref)	1 (ref)	1 (ref)
Black	0.95 (0.89–1.00)	1.00 (0.91–1.11)	1.48 (1.38–1.58)	0.80 (0.72–0.88)	1.16 (0.94–1.43)	1.00 (0.95–1.07)	1.68 (1.57–1.80)	1.48 (1.40–1.56)
Hispanic	1.14 (1.05–1.24)	1.28 (1.12–1.48)	1.01 (0.88–1.16)	0.66 (0.55–0.79)	0.61 (0.37–1.02)	0.96 (0.87–1.07)	1.07 (0.93–1.23)	1.03 (0.92–1.15)
Asian	0.73 (0.65–0.82)	1.80 (1.59 2.04)	0.90 (0.77–1.06)	0.48 (0.38–0.61)	0.72 (0.44–1.16)	1.04 (0.93–1.16)	1.51 (1.33–1.71)	1.00 (0.88–1.14)
Other	1.03 (0.93–1.13)	1.22 (1.04–1.43)	0.94 (0.80–1.09)	0.82 (0.67–1.01)	1.19 (0.81–1.74)	0.94 (0.83–1.06)	1.44 (1.26–1.64)	1.20 (1.07–1.35)
Medicaid buy-in	1.01 (0.96–1.05)	0.84 (0.78–0.90)	0.79 (0.74–0.84)	1.33 (1.25–1.42)	0.67 (0.56–0.80)	0.97 (0.93–1.02)	0.95 (0.90–1.01)	0.88 (0.83–0.92)
Diabetes	1.35 (1.30–1.39)	1.01 (0.95–1.08)	0.82 (0.78–0.87)	1.25 (1.18–1.33)	0.58 (0.48–0.69)	0.91 (0.88–0.95)	0.83 (0.78–0.88)	1.06 (1.01–1.10)
Coronary heart disease	0.86 (0.82–0.90)	0.75 (0.69–0.82)	0.60 (0.55–0.65)	1.12 (1.05–1.20)	0.63 (0.51–0.79)	1.74 (1.67–1.81)	0.83 (0.77–0.89)	0.96 (0.91–1.01)
Stroke	1.12 (1.05–1.19)	0.73 (0.64–0.83)	0.60 (0.53–0.68)	1.13 (1.03–1.24)	0.71 (0.52–0.97)	1.12 (1.05–1.19)	1.25 (1.16–1.36)	1.08 (1.01–1.16)
Chronic kidney disease	0.79 (0.74–0.84)	0.73 (0.65–0.81)	0.62 (0.55–0.69)	1.19 (1.10–1.28)	0.64 (0.48–0.86)	1.20 (1.14–1.27)	1.47 (1.37–1.57)	1.06 (1.00–1.13)
Heart failure	0.90 (0.85–0.96)	0.72 (0.65–0.81)	0.54 (0.48–0.61)	3.23 (3.03 3.45)	0.52 (0.38–0.71)	1.25 (1.19–1.32)	0.85 (0.78–0.92)	1.45 (1.37–1.53)

Abbreviations: ACE =  angiotensin-converting-enzyme; ARB =  Angiotensin receptor blocker; CCB =  Calcium channel blocker. The comparison group for each medication class initiated is not initiating that medication class.

The comparison group for initiating more than one antihypertensive medication class is initiating only one medication class.

Drug initiation patterns were similar when limited to beneficiaries without any compelling indications (Table S2). Specifically, in both the overall pooled cohort and among those without any compelling indications, initiation with a thiazide-type diuretic was less common in later study years, and more common in younger, female, and black beneficiaries. One notable difference is that in the overall cohort there was no time trend for initiation with a loop diuretic, but among those without compelling indications loop diuretics were more commonly initiated in later years.

Among beneficiaries in the pooled 2007–2010 cohort, 19,891 (61.9%) initiated antihypertensive treatment with a medication class recommended by JNC 8. Among beneficiaries without CKD, 48.8% of blacks and 66.7% of non-blacks initiated antihypertensive medication with a drug class recommended by JNC 8. Among beneficiaries with CKD, 34.3% initiated antihypertensive medication with an ACE-I or ARB (30.7% of blacks and 34.9% of non-blacks).

## Discussion

In the current study of a national sample of older hypertensive US adults we found ACE-Is to be the most commonly initiated class of antihypertensive medication, followed by beta blockers, thiazide-type diuretics, CCBs, and ARBs. Initiation of antihypertensive medication with a thiazide-type diuretic decreased from 19.2% in 2007 to 17.9% in 2010. No other changes in initiation rates by class occurred between 2007 and 2010. Younger age, female sex, and black race were each associated with a higher likelihood for initiating antihypertensive treatment with a thiazide-type diuretic. However, initiating antihypertensive treatment with a thiazide-type diuretic was uncommon (<30%) in all of the subgroups investigated. Additionally, the percent of beneficiaries initiating antihypertensive treatment with multiple medication classes declined over time from 25.6% in 2007 to 24.1% in 2010.

In 2003, JNC 7 recommended thiazide-type diuretics for patients with uncomplicated hypertension initiating antihypertensive treatment [2]. Several studies have found that initiation of antihypertensive treatment with a thiazide-type diuretic increased after the publication of JNC 7 [3]–[5]. For example, an analysis of data from a national network of clinics in the US found that the percentage of patients initiating antihypertensive medication with a thiazide-type diuretic increased from 29% to 39% after publication of JNC 7 [5]. However, a more recent study in a separate national network of clinics found that only 16% of patients on antihypertensive monotherapy initiated with a thiazide-type diuretic [17]. Results of the current analysis are consistent with the published literature in suggesting that despite the recommendations of the JNC 7 guideline, the majority of US patients do not initiate antihypertensive treatment with a thiazide-type diuretic. Data from the current study also suggest that the proportion of patients initiating antihypertensive medication with a thiazide-type diuretic has decreased modestly in recent years, despite an initial increase following publication of JNC 7. The current study found that the percentages of patients initiating antihypertensive treatment with an ACE-I or CCB were about 31% and 17%, respectively, similar to what has been reported in literature examining initiation rates in the early 2000s [4], [5].

Randomized controlled trials following the publication of ALLHAT and JNC 7 that compared cardiovascular risk reduction associated with taking CCBs, ACE-Is, and thiazide-type diuretics have produced mixed results [8]–[11]. For example, in 2008 a large randomized trial conducted in a high cardiovascular risk hypertensive population reported that combination therapy with an ACE-I and a CCB was superior to the same ACE-I combined with a thiazide-type diuretic in preventing the composite outcome of death from cardiovascular causes, nonfatal myocardial infarction, nonfatal stroke, hospitalization for angina, resuscitation after sudden cardiac arrest, and coronary revascularization (hazard ratio: 0.80; 95% CI: 0.72–0.90) [8]. However, other trials did not find differences in cardiovascular risk associated with ACE-Is [11] or CCBs [10] versus thiazide-type diuretics. In a 2009 pooled analysis of 46 randomized control trials comparing beta blockers, ACE-Is, ARBs, CCBs and thiazide-type diuretics, no drug classes were found to be more effective than others in reducing the incidence of CHD [9]. While CCBs were more effective in preventing stroke compared to other drug classes (relative risk: 0.91; 95% CI: 0.84–0.98), they were less effective in preventing heart failure (relative risk: 1.22; 95% CI: 1.10–1.35). Despite these data, between 2007 and 2010 we found a slight decline in thiazide-type diuretic initiation but did not find a corresponding trend of increasing initiation with an ACE-I or CCB.

Several recent studies have examined prevalent use of antihypertensive medication classes among US adults. The US National Health and Nutrition Examination Survey (NHANES) provide nationally representative data for prevalent antihypertensive medication use. In an analysis of serial NHANES, the overall percentage of individuals with hypertension taking thiazide-type diuretics rose from 22% in 2001–2002 to 28% in 2009–2010 [18]. However, this analysis also found that the percentage of individuals taking ACE-Is, beta blockers, CCBs, or ARBs all rose over the past decade. In addition, antihypertensive polypharmacy has increased over the past decade. Between 2001–2002 and 2009–2010, the percent of US adults with hypertension taking 2 or more classes of antihypertensive medications increased from 37% to 48% [18]. The current study found that the percentage of older US adults initiating antihypertensive treatment with 2 or more classes of medication decreased, albeit modestly, between 2007 and 2010. In the context of the published literature on prevalent antihypertensive medication use, the findings from the current study suggest that patients are increasingly initiating antihypertensive medication with a single class and are subsequently up-titrated to a regimen that includes multiple classes of antihypertensive medication to control blood pressure. This interpretation is supported by a recent study which found that, following publication of JNC 7, patients were initiating antihypertensive medications at a lower systolic and diastolic blood pressure threshold, and thus were less likely to have an indication for multiple classes of medications at the start of therapy [19].

The current study provides data on antihypertensive medication initiation patterns among older US adults in the time period between publication of the JNC 7 and JNC 8 guidelines. Whereas JNC 7 recommended a thiazide-type diuretic as first-line therapy for newly diagnosed patients with hypertension and no other compelling indications, the recently published JNC 8 guideline suggests that in the general nonblack population, including those with diabetes, the initial antihypertensive treatment regimen should include a thiazide-type diuretic, CCB, ACE-I, or ARB. In the general black population, initial antihypertensive treatment should include a thiazide-type diuretic or CCB. Other classes of antihypertensive medication (e.g., beta blockers, alpha blockers) are not recommended for use as first-line antihypertensive therapy in the JNC 8 guideline. Whereas JNC 7 listed several comorbidities that were compelling indications for specific medication classes, JNC 8 only recommended an ACE-I or ARB for those with CKD. We found that 61.9% of Medicare beneficiaries initiated antihypertensive medication in 2007–2010 with an antihypertensive medication class recommended by JNC 8. While this compares favorably to studies that have consistently shown that <40% of patients initiated antihypertensive medication with a thiazide-type diuretic following JNC 7, whether the JNC 8 guideline will affect treatment regimens for patients initiating antihypertensive medication is not yet known and should be investigated in future studies.

Our study has several strengths. We used national data on US adults 65 years of age and older from Medicare. Identifying treatment patterns among older adults is important given their high incidence of hypertension [20] and increased risk for adverse blood pressure-related outcomes including CHD, stroke and end-stage renal disease [21]. Most prior studies have relied on prevalent users of antihypertensive medication. Using Medicare, we were able to assess the initiation of, and secular trends in, antihypertensive medication classes through 2010. This study also has limitations. As with all claims-based analyses, our results depend on the accuracy of claims to identify comorbid conditions and pharmacy fills. While claims-based algorithms for CHD, stroke, diabetes and heart failure have high positive predictive value, algorithms for identifying CKD in Medicare do not [22]. In addition, some beneficiaries may not have submitted claims for reimbursement when initiating antihypertensive medication. However, prior studies suggest that out-of-pocket payments for generic medications among Medicare beneficiaries are not common [23].

## Conclusions

Despite the recommendation in JNC 7, in this nationwide study, less than 1 in 5 Medicare beneficiaries initiated antihypertensive medication with a thiazide-type diuretic. The proportion initiating antihypertensive treatment with a thiazide-type diuretic was higher in those without comorbid conditions, but remained below 30% in every subgroup investigated. These data suggest a disconnect between US national guidelines and clinical practice for the treatment of hypertension. Over 30% of Medicare beneficiaries initiated antihypertensive medication with drug classes not recommended as first line therapy in the 2014 JNC 8 guideline. Effective dissemination efforts for the JNC 8 guideline are needed to ensure that patients receive appropriate antihypertensive treatment.

## Supporting Information

Figure S1 **CONSORT diagram for the analysis Medicare beneficiaries initiating antihypertensive treatment.**
(PNG)

Table S1 **Most common pairs of antihypertensive medication classes initiated in the pooled 2007–2010 5% Medicare sample who initiated >1 antihypertensive medication, by calendar year.**
(DOCX)

Table S2 **Multivariable adjusted risk ratios for initiating each antihypertensive medication class in the pooled 2007–2010 5% Medicare sample without any compelling indication.**
(DOCX)

Appendix S1 **Compelling indication history claims algorithm definitions.**
(DOCX)
