# Correction: Antihypertensive Medication Classes Used among Medicare Beneficiaries Initiating Treatment in 2007–2010

**DOI:** 10.1371/journal.pone.0118763

**Published:** 2015-03-24

**Authors:** The PLOS ONE Staff

There is an error in the CCBs column heading of Table 3; the correct number is 5593. Please see the correct Table 3 here.

**Table 3:** Multivariable adjusted risk ratios and 95% confidence intervals for initiating each antihypertensive medication class among Medicare beneficiaries in 2007–2010.

	Antihypertensive medication class initiated							
Characteristic	ACE-inhibitors (n = 10105)	ARBs (n = 4583)	Thiazide diuretics (n = 6040)	Loop diuretics (n = 3798)	Potassium-sparing diuretics (n = 973)	Beta blockers (n = 8658)	CCBs (n = 5593)	>1 class (n = 7961)
Year								
2007	1 (ref)	1 (ref)	ref	1 (ref)	1 (ref)	1 (ref)	1 (ref)	ref
2008	0.95 (0.91–0.99)	1.03 (0.95–1.11)	0.99 (0.93–1.06)	1.03 (0.95–1.11)	1.07 (0.90–1.27)	1.00 (0.95–1.05)	1.00 (0.93–1.07)	0.97 (0.92–1.02)
2009	0.99 (0.95–1.04)	0.99 (0.91–1.06)	0.98 (0.92–1.04)	1.03 (0.95–1.12)	0.93 (0.78–1.11)	1.00 (0.95–1.06)	0.96 (0.90–1.03)	0.97 (0.92–1.03)
2010	0.98 (0.94–1.03)	0.99 (0.92–1.07)	0.92 (0.86–0.98)	1.03 (0.95–1.12)	0.90 (0.75–1.08)	1.01 (0.96–1.06)	0.98 (0.92–1.05)	0.95 (0.90–1.00)
Age								
65–69	1 (ref)	1 (ref)	1 (ref)	1 (ref)	1 (ref)	1 (ref)	1 (ref)	ref
70–74	0.93 (0.89–0.97)	0.95 (0.88–1.03)	0.92 (0.87–0.98)	1.09 (0.98–1.22)	0.99 (0.82–1.18)	1.02 (0.97–1.08)	1.02 (0.94–1.10)	0.90 (0.85–0.95)
75–79	0.85 (0.81–0.90)	0.96 (0.89–1.04)	0.83 (0.77–0.89)	1.41 (1.27–1.56)	0.93 (0.77–1.13)	1.01 (0.96–1.07)	1.16 (1.07–1.25)	0.89 (0.84–0.95)
80–84	0.79 (0.75–0.84)	0.85 (0.78–0.93)	0.78 (0.73–0.84)	1.69 (1.53–1.88)	0.91 (0.74–1.12)	1.09 (1.03–1.15)	1.11 (1.02–1.20)	0.86 (0.81–0.92)
85+	0.73 (0.70–0.78)	0.72 (0.65–0.79)	0.67 (0.62–0.73)	2.23 (2.02 2.46)	0.88 (0.72–1.08)	1.07 (1.01–1.14)	1.20 (1.11–1.30)	0.82 (0.77–0.87)
Male	1.10 (1.06–1.13)	0.84 (0.79–0.88)	0.82 (0.78–0.86)	1.00 (0.95–1.06)	0.55 (0.47–0.64)	1.01 (0.98–1.05)	0.99 (0.94–1.04)	0.95 (0.92–0.99)
Race/ethnicity								
White	1 (ref)	1 (ref)	ref	1 (ref)	1 (ref)	1 (ref)	1 (ref)	1 (ref)
Black	0.95 (0.89–1.00)	1.00 (0.91–1.11)	1.48 (1.38–1.58)	0.80 (0.72–0.88)	1.16 (0.94–1.43)	1.00 (0.95–1.07)	1.68 (1.57–1.80)	1.48 (1.40–1.56)
Hispanic	1.14 (1.05–1.24)	1.28 (1.12–1.48)	1.01 (0.88–1.16)	0.66 (0.55–0.79)	0.61 (0.37–1.02)	0.96 (0.87–1.07)	1.07 (0.93–1.23)	1.03 (0.92–1.15)
Asian	0.73 (0.65–0.82)	1.80 (1.59 2.04)	0.90 (0.77–1.06)	0.48 (0.38–0.61)	0.72 (0.44–1.16)	1.04 (0.93–1.16)	1.51 (1.33–1.71)	1.00 (0.88–1.14)
Other	1.03 (0.93–1.13)	1.22 (1.04–1.43)	0.94 (0.80–1.09)	0.82 (0.67–1.01)	1.19 (0.81–1.74)	0.94 (0.83–1.06)	1.44 (1.26–1.64)	1.20 (1.07–1.35)
Medicaid buy-in	1.01 (0.96–1.05)	0.84 (0.78–0.90)	0.79 (0.74–0.84)	1.33 (1.25–1.42)	0.67 (0.56–0.80)	0.97 (0.93–1.02)	0.95 (0.90–1.01)	0.88 (0.83–0.92)
Diabetes	1.35 (1.30–1.39)	1.01 (0.95–1.08)	0.82 (0.78–0.87)	1.25 (1.18–1.33)	0.58 (0.48–0.69)	0.91 (0.88–0.95)	0.83 (0.78–0.88)	1.06 (1.01–1.10)
Coronary heart disease	0.86 (0.82–0.90)	0.75 (0.69–0.82)	0.60 (0.55–0.65)	1.12 (1.05–1.20)	0.63 (0.51–0.79)	1.74 (1.67–1.81)	0.83 (0.77–0.89)	0.96 (0.91–1.01)
Stroke	1.12 (1.05–1.19)	0.73 (0.64–0.83)	0.60 (0.53–0.68)	1.13 (1.03–1.24)	0.71 (0.52–0.97)	1.12 (1.05–1.19)	1.25 (1.16–1.36)	1.08 (1.01–1.16)
Chronic kidney disease	0.79 (0.74–0.84)	0.73 (0.65–0.81)	0.62 (0.55–0.69)	1.19 (1.10–1.28)	0.64 (0.48–0.86)	1.20 (1.14–1.27)	1.47 (1.37–1.57)	1.06 (1.00–1.13)
Heart failure	0.90 (0.85–0.96)	0.72 (0.65–0.81)	0.54 (0.48–0.61)	3.23 (3.03 3.45)	0.52 (0.38–0.71)	1.25 (1.19–1.32)	0.85 (0.78–0.92)	1.45 (1.37–1.53)

Abbreviations: ACE = angiotensin-converting-enzyme; ARB = Angiotensin receptor blocker; CCB = Calcium channel blocker.

The comparison group for each medication class initiated is not initiating that medication class.

The comparison group for initiating more than one antihypertensive medication class is initiating only one medication class.
