# Supplementary figures and images for: Antihypertensive Medication Classes Used among Medicare Beneficiaries Initiating Treatment in 2007–2010
Source: PLoS One. 2014 Aug 25;9(8):e105888. doi: 10.1371/journal.pone.0105888 (PMC4143342; doi:10.1371/journal.pone.0105888)

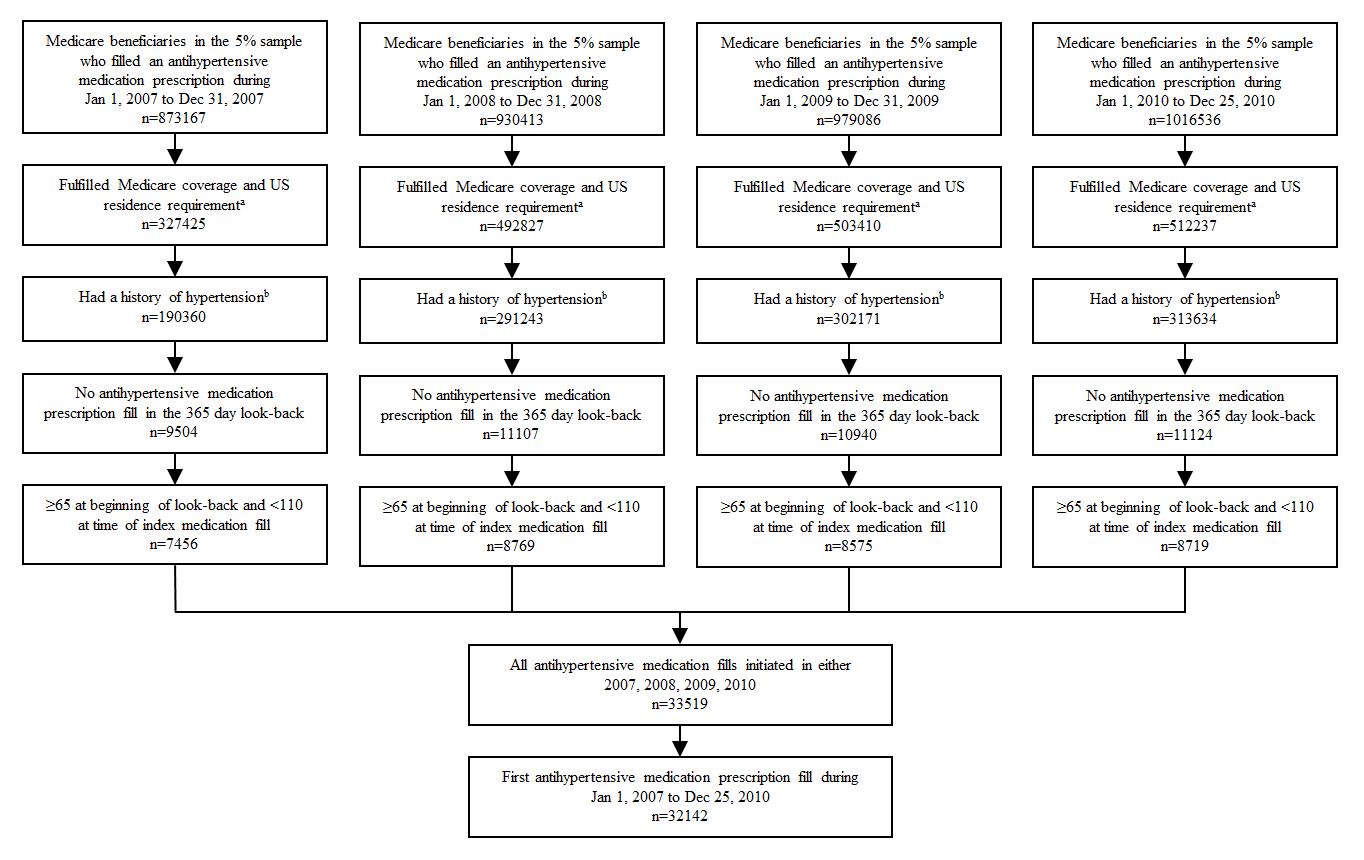

Supplement: Figure S1 — CONSORT diagram for the analysis Medicare beneficiaries initiating antihypertensive treatment. (PNG) [file pone.0105888.s001.png]
