# Supplementary material for: Antihypertensive Medication Classes Used among Medicare Beneficiaries Initiating Treatment in 2007–2010
Source: PLoS One. 2014 Aug 25;9(8):e105888. doi: 10.1371/journal.pone.0105888 (PMC4143342; doi:10.1371/journal.pone.0105888)
Supplement: Table S1 — Most common pairs of antihypertensive medication classes initiated in the pooled 2007–2010 5% Medicare sample who initiated >1 antihypertensive medication, by calendar year. (DOCX) [file pone.0105888.s002.docx]

Table S1. Most common pairs of antihypertensive medication classes initiated in the pooled 2007-2010 5% Medicare sample who initiated >1 antihypertensive medication, by calendar year

|  | Calendar Year (Eligible Population) | | | |
| --- | --- | --- | --- | --- |
| Antihypertensive medication classes | 2007 (n=1908) | 2008 (n=2163) | 2009 (n=2090) | 2010 (n=2102) |
| Thiazide diuretic and ACE-inhibitor | 375 (19.7%) | 467 (21.6%) | 501 (24.0%) | 476 (22.6%) |
| Thiazide diuretic and ARB | 396 (20.8%) | 455 (21.0%) | 407 (19.5%) | 404 (19.2%) |
| ACE-inhibitor and Beta blocker | 312 (16.4%) | 364 (16.8%) | 365 (17.5%) | 356 (16.9%) |
| ACE-inhibitor and CCB | 365 (19.1%) | 288 (13.3%) | 272 (13.0%) | 239 (11.4%) |
| Thiazide diuretic and Beta blocker | 185 (9.7%) | 183 (8.5%) | 194 (9.3%) | 190 (9.0%) |
| Beta blocker and CCB | 156 (8.2%) | 198 (9.2%) | 172 (8.2%) | 179 (8.5%) |
| Beta blocker and Loop diuretic | 179 (9.4%) | 186 (8.6%) | 173 (8.3%) | 171 (8.1%) |
| ACE-inhibitor and Loop diuretic | 140 (7.3%) | 150 (6.9%) | 117 (5.6%) | 154 (7.3%) |
| CCB and ARB | 55 (2.9%) | 114 (5.3%) | 113 (5.4%) | 150 (7.1%) |
| Thiazide diuretics and CCB | 88 (4.6%) | 120 (5.5%) | 112 (5.4%) | 106 (5.0%) |

Note: Number of antihypertensive medications initiated are not mutually exclusive; column percentages add to >100%.

Note: Classes initiated by fewer than 100 beneficiaries are not shown

Abbreviations: ACE= Angiotensin-converting-enzyme; ARB= Angiotensin receptor blocker; CCB= Calcium channel blocker
