# Supplementary material for: Antihypertensive Medication Classes Used among Medicare Beneficiaries Initiating Treatment in 2007–2010
Source: PLoS One. 2014 Aug 25;9(8):e105888. doi: 10.1371/journal.pone.0105888 (PMC4143342; doi:10.1371/journal.pone.0105888)
Supplement: Table S2 — Multivariable adjusted risk ratios for initiating each antihypertensive medication class in the pooled 2007–2010 5% Medicare sample without any compelling indication. (DOCX) [file pone.0105888.s003.docx]

Table S2. Multivariable adjusted risk ratios for initiating each antihypertensive medication class in the pooled 2007-2010 5% Medicare sample without any compelling indication^†^.

|  | Antihypertensive medication initiated | | | | | | | |
| --- | --- | --- | --- | --- | --- | --- | --- | --- |
| Characteristic | ACE-inhibitors (n=4917) | ARBs (n=2662) | Thiazide diuretics (n=3942) | Loop diuretics (n=1263) | Potassium-sparing diuretics (n=678) | Beta blockers (n=3702) | CCBs (n=2944) | >1 class (n=3856) |
| Year |  |  |  |  |  |  |  |  |
| 2007 | ref | ref | ref | ref | ref | ref | ref | ref |
| 2008 | 0.95 (0.89-1.01) | 1.02 (0.92-1.12) | 0.99 (0.92-1.07) | 1.12 (0.96-1.30) | 1.03 (0.84-1.25) | 0.98 (0.91-1.07) | 1.04 (0.95-1.14) | 0.98 (0.91-1.06) |
| 2009 | 1.00 (0.94-1.07) | 0.97 (0.88-1.07) | 0.99 (0.92-1.07) | 1.09 (0.93-1.27) | 0.90 (0.73-1.11) | 0.97 (0.90-1.06) | 1.00 (0.91-1.10) | 0.96 (0.89-1.04) |
| 2010 | 0.99 (0.93-1.06) | 0.97 (0.87-1.07) | 0.91 (0.84-0.98) | 1.23 (1.05-1.43) | 0.84 (0.67-1.04) | 1.03 (0.95-1.11) | 1.02 (0.93-1.12) | 0.95 (0.88-1.03) |
| Age(years) |  |  |  |  |  |  |  |  |
| 65-69 | ref | ref | ref | ref | ref | ref | ref | ref |
| 70-74 | 0.91 (0.85-0.96) | 0.99 (0.90-1.09) | 0.91 (0.84-0.98) | 1.13 (0.92-1.39) | 0.96 (0.78-1.19) | 1.10 (1.01-1.20) | 1.00 (0.91-1.11) | 0.90 (0.83-0.97) |
| 75-79 | 0.82 (0.77-0.88) | 0.98 (0.88-1.08) | 0.81 (0.75-0.88) | 1.73 (1.43 2.10) | 0.97 (0.78-1.21) | 1.09 (1.00-1.19) | 1.19 (1.07-1.31) | 0.88 (0.81-0.96) |
| 80-84 | 0.79 (0.73-0.85) | 0.90 (0.80-1.00) | 0.78 (0.71-0.85) | 2.31 (1.91 2.79) | 0.88 (0.69-1.12) | 1.14 (1.04-1.25) | 1.17 (1.05-1.30) | 0.85 (0.78-0.93) |
| 85+ | 0.71 (0.65-0.77) | 0.70 (0.62-0.79) | 0.66 (0.60-0.72) | 3.33 (2.78 3.99) | 0.82 (0.64-1.05) | 1.16 (1.05-1.27) | 1.26 (1.14-1.40) | 0.78 (0.71-0.85) |
| Male | 1.14 (1.09-1.20) | 0.86 (0.80-0.93) | 0.82 (0.77-0.87) | 0.95 (0.85-1.07) | 0.52 (0.43-0.63) | 1.01 (0.95-1.08) | 1.06 (0.99-1.13) | 1.00 (0.95-1.07) |
| Race/ethnicity |  |  |  |  |  |  |  |  |
| White | ref | ref | ref | ref | ref | ref | ref | ref |
| Black | 0.94 (0.86-1.03) | 0.88 (0.76-1.02) | 1.44 (1.32-1.57) | 0.73 (0.59-0.90) | 1.18 (0.90-1.54) | 0.91 (0.82-1.02) | 1.76 (1.60-1.94) | 1.54 (1.41-1.68) |
| Hispanic | 1.04 (0.91-1.20) | 1.42 (1.18-1.71) | 1.17 (0.99-1.38) | 0.55 (0.39-0.78) | 0.71 (0.39-1.30) | 0.85 (0.71-1.02) | 1.08 (0.88-1.33) | 1.09 (0.92-1.29) |
| Asian | 0.62 (0.52-0.75) | 1.76 (1.50 2.07) | 0.84 (0.68-1.02) | 0.34 (0.22-0.54) | 0.77 (0.43-1.37) | 0.97 (0.81-1.15) | 1.58 (1.33-1.86) | 0.93 (0.77-1.12) |
| Other | 0.96 (0.82-1.12) | 1.19 (0.96-1.49) | 1.07 (0.89-1.28) | 0.61 (0.39-0.94) | 1.30 (0.82 2.05) | 0.93 (0.76-1.13) | 1.40 (1.15-1.70) | 1.17 (0.99-1.40) |
| Medicaid buy-in | 0.98 (0.92-1.05) | 0.86 (0.78-0.95) | 0.78 (0.72-0.84) | 1.84 (1.63 2.09) | 0.62 (0.49-0.77) | 1.03 (0.95-1.11) | 0.97 (0.89-1.06) | 0.86 (0.80-0.93) |

Abbreviations: ACE= angiotensin-converting-enzyme; ARB=Angiotensin receptor blocker; CCB= Calcium channel blocker.

†Compelling indication is defined by a beneficiary having diabetes, coronary heart disease, stroke, chronic kidney disease, or heart failure.

Risk ratios are shown with 95% confidence intervals.

Each medication class was compared with initiating antihypertensive medication with other classes (e.g., the outcome for the ACE inhibitor column was filling an ACE inhibitor versus filling other classes of antihypertensive medication).

Initiating more than one antihypertensive medication class was compared with initiating only one medication class.
