# Supplementary material for: Antihypertensive Medication Classes Used among Medicare Beneficiaries Initiating Treatment in 2007–2010
Source: PLoS One. 2014 Aug 25;9(8):e105888. doi: 10.1371/journal.pone.0105888 (PMC4143342; doi:10.1371/journal.pone.0105888)
Supplement: Appendix S1 — Compelling indication history claims algorithm definitions. (DOCX) [file pone.0105888.s004.docx]

Appendix S1. Compelling indication history claims algorithm definitions

*History of diabetes mellitus* [[1](#_ENREF_1)]

Any one of the following:

1. At least 1 inpatient claim with discharge ICD-9 diagnoses (any position) of 250.xx, 357.2, 362.0x, or 366.41
2. At least 2 carrier claim, carrier line or outpatient claims with ICD-9 diagnoses (any position) of 250.xx, 357.2, 362.0x, or 366.41, linked by CLAIM_ID to an ambulatory physician evaluation and management claim, with the 2 claims occurring at least 7 days apart
3. At least 1 prescription record for an oral antidiabetes medication or insulin fills (using national medication codes from Supplemental Table 2).

*History of coronary heart disease* [[2](#_ENREF_2)]

Any one of the following:

1. Acute myocardial infarction: At least 1 inpatient claim with discharge ICD-9 diagnoses (any position) of 410 (3-digit substring)
2. Coronary revascularization: At least 1 inpatient or carrier claim, carrier line or outpatient claim with CPT codes 92980-92996 (angioplasty or stent) or 33510-33536 (coronary artery bypass graft) or ICD-9 procedure codes 00.66 or 36.01-36.09 (angioplasty or stent) or 36.10-36.19 (coronary artery bypass graft)
3. Other ischemic heart disease: Either one of the following:
   1. At least 1 inpatient claim with ICD-9 diagnoses (any position) of 411.xx, 412.00, 413.xx, or 414.xx
   2. At least 2 carrier claim, carrier line or outpatient claims with ICD-9 diagnoses (any position) of 411.xx, 412.00, 413.xx, or 414.xx, linked by CLAIM_ID to an ambulatory physician evaluation and management claim, with the 2 claims occurring at least 7 days apart

*History of stroke* [[3](#_ENREF_3)]

Any one of the following:

1. At least 1 inpatient ICD-9 diagnosis (any position) of 430.xx, 431.xx, 433.x1, 434.x1 or 436.x
2. At least 1 carrier claim, carrier line or outpatient claims with ICD-9 diagnoses (any position) of 430.xx, 431.xx, 433.x1, 434.x1 or 436.x, linked by CLAIM_ID to an ambulatory physician evaluation and management claim
3. At least 1 claim with ICD-9 diagnoses (any position) of 430.xx, 431.xx, 433.x1, 434.x1 or 436.x in other file types (home health aide, durable medical equipment, hospice, skilled nursing facility)

*History of chronic kidney disease* [[4](#_ENREF_4)]

Any one of the following:

1. at least 1 inpatient claim with discharge ICD-9 kidney disease diagnoses: 016.0, 095.4, 189.0, 189.9, 223.0, 236.91, 250.4, 271.4, 274.1, 283.11, 403.xx, 404.xx, 440.1, 442.1, 447.3, 572.4, 580.xx–588.xx, 591, 642.1, 646.2, 753.12–753.17, 753.19, 753.2, 794.4
2. at least 2 carrier claim, carrier line or outpatient claims with kidney disease ICD-9 diagnoses above (any position) , with the 2 claims occurring at least 7 days apart.

*History of heart failure* [[2](#_ENREF_2)]

At least one inpatient or outpatient, or carrier line or claim (any position) linked by CLAIM_ID to an ambulatory physician evaluation and management claim with ICD-9 diagnoses of 398.91, 402.01, 402.11, 402.91, 404.01, 404.11, 404.91, 404.03, 404.13, 404.93, 428.0, 428.1, 428.20, 428.21, 428.22, 428.23, 428.30, 428.31, 428.32, 428.33, 428.40, 428.41, 428.42, 428.43, or 428.9

REFERENCES

1. Miller DR, Safford MM, Pogach LM (2004) Who has diabetes? Best estimates of diabetes prevalence in the Department of Veterans Affairs based on computerized patient data. Diabetes Care 27 Suppl 2: B10-21.

2. Centers for Medicare and Medicaid Services Chronic conditions data warehouse codition categories.

3. Graham DJ, Ouellet-Hellstrom R, MaCurdy TE, Ali F, Sholley C, et al. (2010) Risk of acute myocardial infarction, stroke, heart failure, and death in elderly Medicare patients treated with rosiglitazone or pioglitazone. JAMA 304: 411-418. doi: 10.1001/jama.2010.920

4. U.S. Renal Data System Coordinating Center (2012) USRDS 2012 Researcher’s Guide to the USRDS Database. 39 p.
